# Selective sphingosine-1-phosphate receptor 1 modulator attenuates blood–brain barrier disruption following traumatic brain injury by inhibiting vesicular transcytosis

**DOI:** 10.1186/s12987-022-00356-6

**Published:** 2022-07-11

**Authors:** Yuan Zhang, Lin Wang, Qiuling Pan, Xiaomin Yang, Yunchuan Cao, Jin Yan, Yingwen Wang, Yihao Tao, Runjin Fan, Xiaochuan Sun, Lin Li

**Affiliations:** 1 Department of Neurosurgery, Neural Injury and Protection Laboratory, The First Affiliated Hospital of Chongqing Medical University, Chongqing, 400016 China; 2 Department of Neurosurgery, Nanchong Central Hospital, The Second Clinical Medical College of North Sichuan Medical College, Nanchong, China; 3 Department of Neurosurgery, The Second Affiliated Hospital of Chongqing Medical University, Chongqing, China

**Keywords:** Traumatic brain injury, Blood–brain barrier, Vesicular transcytosis, Mfsd2a, S1P1 modulator

## Abstract

**Background:**

Traumatic brain injury (TBI) provokes secondary pathological damage, such as damage to the blood–brain barrier (BBB), ischaemia and inflammation. Major facilitator superfamily domain-containing 2a (Mfsd2a) has been demonstrated to be critical in limiting the increase in BBB vesicle transcytosis following brain injury. Recent studies suggest that a novel and selective modulator of the sphingosine-1-phosphate receptor 1 (S1P1), CYM-5442, maintains the integrity of the BBB by restricting vesicle transcytosis during acute ischaemic stroke. In the current study, we investigated whether CYM-5442, evaluated in a short-term study, could protect the brains of mice with acute-stage TBI by reversing the increase in vesicle transport due to reduced Mfsd2a expression after TBI.

**Methods:**

We used the well-characterized model of TBI caused by controlled cortical impact. CYM-5442 (0.3, 1, 3 mg/kg) was intraperitoneally injected 30 min after surgery for 7 consecutive days. To investigate the effect of CYM-5442 on vesicle transcytosis, we downregulated and upregulated Mfsd2a expression using a specific AAV prior to evaluation of the TBI model. MRI scanning, cerebral blood flow, circulating blood counts, ELISA, TEM, WB, and immunostaining evaluations were performed after brain injury.

**Results:**

CYM-5442 significantly attenuated neurological deficits and reduced brain oedema in TBI mice. CYM-5442 transiently suppressed lymphocyte trafficking but did not induce persistent lymphocytopenia. After TBI, the levels of Mfsd2a were decreased significantly, while the levels of CAV-1 and albumin were increased. In addition, Mfsd2a deficiency caused inadequate sphingosine-1-phosphate (S1P) transport in the brain parenchyma, and the regulation of BBB permeability by Mfsd2a after TBI was shown to be related to changes in vesicle transcytosis. Downregulation of Mfsd2a in mice markedly increased the BBB permeability, neurological deficit scores, and brain water contents after TBI. Intervention with CYM-5442 after TBI protected the BBB by significantly reducing the vesicle transcytosis of cerebrovascular endothelial cells.

**Conclusion:**

In addition to transiently suppressing lymphocytes, CYM-5442 alleviated the neurological deficits, cerebral edema and protective BBB permeability in TBI mice by reducing the vesicle transcytosis of cerebrovascular endothelial cells.

**Supplementary Information:**

The online version contains supplementary material available at 10.1186/s12987-022-00356-6.

## Introduction

TBI is a leading cause of death and disability in children and adolescents [1] and has become a critical global public health and socioeconomic issue [2]. TBI accounts for 25–33% of all accidental deaths, and approximately two-thirds of hospital deaths are associated with trauma [3, 4]. TBI has high rates of mortality and morbidity and imposes a serious public health threat. Therefore, it is particularly important to elucidate its underlying pathological mechanism and identify reliable therapeutic strategies. Although great efforts have been made to identify treatment options for TBI and its secondary complications, their efficacies remains far from ideal [5]. In a series of pathological changes after TBI, breakdown of the BBB is considered to be a key factor determining the severity and recovery time of craniocerebral injury [6–8]. Therefore, it is particularly important to investigate the potential mechanism of vascular endothelial function and BBB permeability after TBI.

Theoretically, BBB permeability is strictly regulated by the paracellular pathway (alterations in tight junction (TJ) function) and/or the transcellular route (vesicular transcytosis)[9]. Previous studies on BBB permeability changes after TBI have mainly focused on damage to TJ complexes via the paracellular pathway [10, 11], while the transcellular pathway has been ignored. Therefore, the role of vesicular transcytosis in brain injury after TBI still needs further study. Mfsd2a is selectively expressed in the cerebrovascular endothelium and acts as a key regulator of BBB permeability by inhibiting the vesicular transcytosis of endothelial cells (ECs) [12]. Previous studies have found that Mfsd2a overexpression after brain injury has a neuroprotective effect by inhibiting vesicular transcytosis [13–15], but whether Mfsd2a plays a role in BBB disruption after TBI remains unknown.

S1P is a signalling lipid that plays a key role in immunity and vascular function through five cognate G protein-coupled receptors (GPCRs), S1P1-5 [16]. The high efficacy of sphingosine 1-phosphate receptor (S1PR) modulators in multiple sclerosis (MS), achieved via the regulation of S1P1, as well as extensive preclinical evidence of their effects in other diseases, supports their use as treatment options for a wide range of diseases [17]. As a basic pathological change, BBB disruption after brain injury further leads to the migration and infiltration of leukocytes into the central nervous system (CNS), thereby increasing the release of various cytokines, proteases and chemokines [18]. Emerging evidence supports that secondary inflammation aggravates brain injury, and inhibition of neuroinflammation after TBI can protect the BBB and provide strategies for post-TBI treatment [19, 20]. Although previous studies have shown that the S1PR modulator FTY-720 plays a key role in a variety of neurological disorders, adverse side effects have been reported due to its nonspecificity [17], and studies have shown that FTY-720 not only does not protect against TBI [21] but also damages the BBB [22]. Unlike first-generation S1P modulators, CYM-5442 is an S1P1-selective agonist that has been reported to be rapidly and preferentially distributed to the brain after systemic injection [23] and is promising for the treatment of ischaemia [24] and influenza viruses [25] by reducing cerebrovascular EC vesicular transport and suppressing the immune system. Previous studies have shown that Mfsd2a interacts with Spinster Homolog 2 (SPNS2) to export S1P across brain ECs, thereby regulating the formation and maintenance of the BBB [22]. Since the expression of Mfsd2a may decrease in the acute stage of brain injury [13–15], intervention with an S1P1 modulator after TBI may protect the BBB.

Although CYM-5442, a specific S1P1 agonist, may play a protective role after brain injury, its role in TBI remains unclear. In this study, we determined the role of Mfsd2a in BBB changes after TBI. Furthermore, a mouse model of TBI was used to evaluate whether intervention with CYM-5442 after TBI could not only inhibit neuroinflammation but also protect the BBB by inhibiting the vesicular transcytosis of cerebrovascular ECs.

## Materials and methods

### Study design

**Fig. 1:**
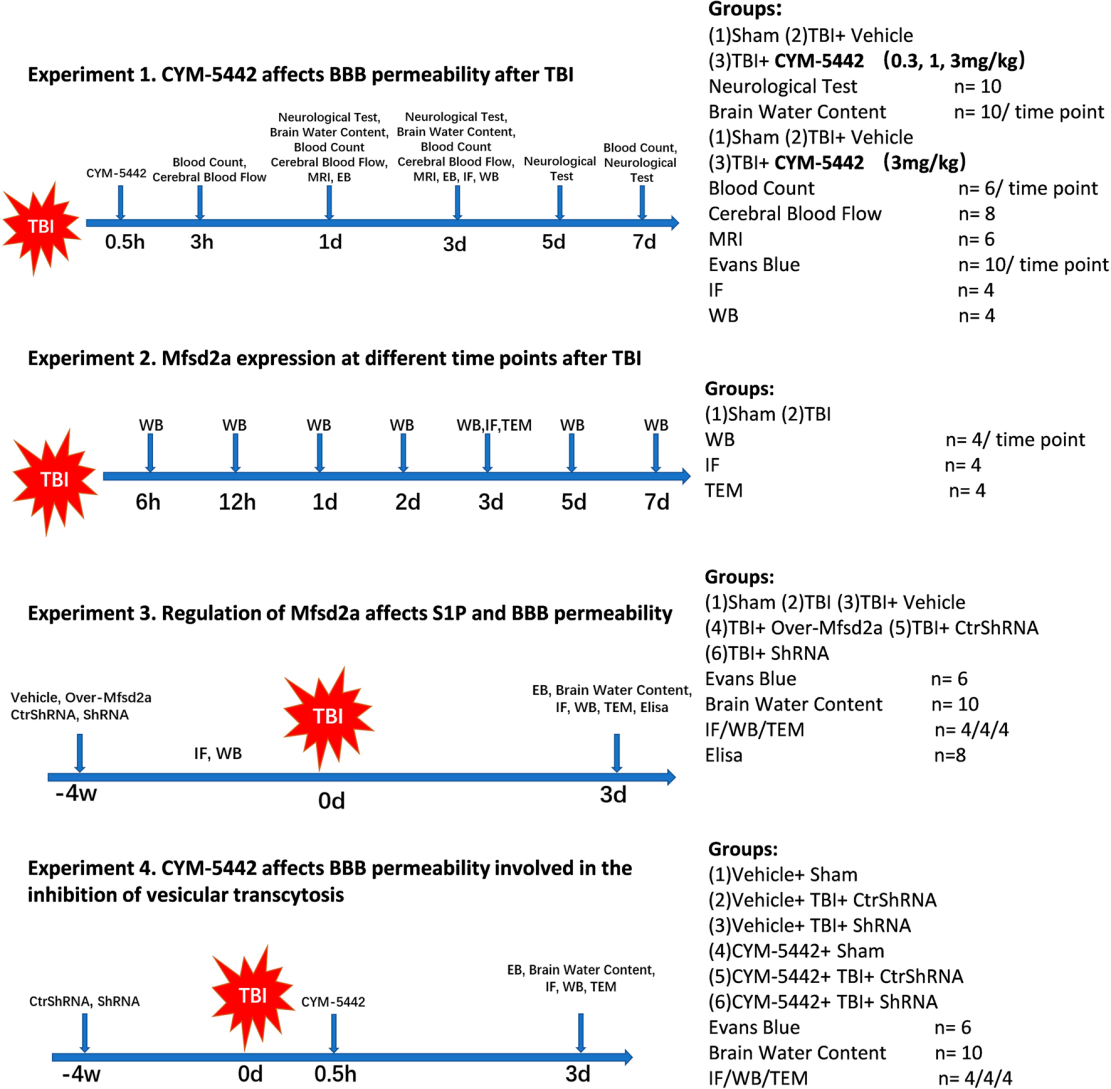
Experimental design and animal grouping. *TBI* traumatic brain injury, *WB* Western blot, *IF* immunofluorescence, *TEM* transmission electron microscopy

In the current study, all the mice were randomly assigned to undergo one of the following four experimental procedures in a blinded manner. The number and distribution of animals in each experimental group are shown in Fig. 1.

### Experiment 1

To determine the effects of CYM-5442 after TBI, mice were assigned to one of three groups: sham, TBI + vehicle, and TBI + CYM-5442. CYM-5442 was dissolved in 0.9% saline containing 10% dimethylsulfoxide (DMSO) and 10% Tween-80 and administered daily intraperitoneally. Vehicle animals received the same volume of saline containing 10% DMSO and 10% Tween-80. Neurological outcomes were evaluated at the corresponding time points following TBI before the animals were sacrificed for sample collection. To determine the therapeutic dosage, mice were treated with 0.3, 1 or 3 mg/kg CYM-5442 at 30 min after TBI induction and administered daily intraperitoneally according to their brain water content and neurobehavioural test results. A final CYM-5442 dosage of 3 mg/kg, administered after TBI, was finally adopted, and Western blot (WB), IF, MRI, blood count and other evaluations were performed.

### Experiment 2

Endogenous expression of Mfsd2a, CAV-1, and claudin-5 was evaluated by WB using samples obtained from the pericontusional cortex at the indicated time point after TBI. Brain ultrastructure was detected by transmission electron microscopy (TEM) at 72 h following TBI. The cellular colocalization of Mfsd2a with an EC marker (CD31) in the pericontusional region was evaluated by double-IF staining (IF) at 72 h following TBI.

### Experiment 3

To enhance or downregulate the protein expression of Mfsd2a, experiment 3 was designed as follows. All mice were randomly divided into 6 groups, the sham, TBI, TBI + Vehicle, TBI + Over-Mfsd2a, TBI + Control-ShRNA and TBI + ShRNA-Mfsd2a groups. All AAVs were administered by intraventricular injection at 4 W before TBI. The mice were euthanized with CO_2_ on the third day after TBI, and their brain tissues were harvested for analysis.

### Experiment 4

The potential mechanism underlying the protective effects of CYM-5442 on BBB integrity through Mfsd2a/CAV-1 was evaluated. The experimental design for the fourth experiment was as follows. All mice were randomly divided into the following six groups: the Vehicle + Sham, Vehicle + TBI + Control-ShRNA, Vehicle + TBI + ShRNA-Mfsd2a, CYM-5442 + Sham, CYM-5442 + TBI + Control-ShRNA and CYM-5442 + TBI + ShRNA-Mfsd2a groups. The mice were euthanized with CO_2_ on the third day after TBI, and their brain tissues were harvested for analysis.

### Study approval

All experiments involving animals were approved by the Chongqing Medical University Administrative Panel on Laboratory Animal Care.

### Animals

Male C57BL/6 mice (8–10 weeks old, 22–26 g) were purchased from the Experimental Animal Center of Chongqing Medical University. The animals were housed on a 12 h light–dark cycle at a controlled room temperature (23 ± 2 °C) and relative humidity (40–60%) and had ad libitum access to food and water. The mice were generally anaesthetized via the intraperitoneal (i.p.) injection of chloral hydrate (3.5%, 350 mg/kg). All surgeries were performed under anaesthesia, and all efforts were made to minimize animal suffering.

### TBI induction

A standard protocol and a controlled cortical impact (CCI) device (TBI-0310, Precision Systems and Instrumentation, Fair fax, VA, USA) were utilized to induce brain injuries as described previously [10]. Briefly, after anaesthetization, a circular craniotomy (3 mm in diameter) was performed (1 mm posterior to bregma and 1 mm lateral to the sagittal suture over the right parietal cortex). Following the craniotomy, the CCI model was established with a TBI-0310 TBI model system with the following impact parameters: velocity, 5.0 m/s; depth, 2.0 mm; and dwelling time, 100 ms. As a result, a moderately severe contusion was induced in the right sensorimotor cortex underlying the hippocampus. The mice exhibited pronounced behavioural deficits but none died [26]. Mice in the Sham group underwent identical surgical procedures but without impact. Following the injury, the hole was sealed with bone wax, and the skin incision was sutured. Finally, the mice were kept on an electric blanket to maintain their normal body temperature until the completely awakened.

### Laser speckle contrast imaging (LSCI)

Cerebral cortical blood flow was monitored at baseline and at 3, 24, and 72 h post-TBI using laser speckle techniques as described previously [10]. Mice were anaesthetized by isoflurane, and an incision was made along the midline to separate the skin from the skull. The mouse body temperature was maintained at 37° ± 0.5 °C throughout the experiment. The exposure area was kept clean and dry using a tampon during image collection. The PeriCam PSI head was adjusted to ensure that the red cross (indicator laser, 660 nm) was located at the centre of the brain, and the measurement distance was kept at 10 cm. The test area was adjusted with PIM Software version 1.5. Cerebral blood signals were collected at 785 nm and translated into blood perfusion images via PIMSoft. Perfusion images were collected with a PeriCam high-resolution LSCI instrument (PSI System, Perimed) with a 70 mW built-in laser diode for illumination and a CCD camera installed 10 cm above the skull. Laser speckle blood flow images were recorded and used to identify the regions of interest (ROIs). The cerebral blood flow (CBF) changes are presented as the mean perfusion values.

### Magnetic resonance imaging

Serial MRI scanning was used to assess lesion volumes and brain oedema on a 7.0 T animal scanner (Bruker Biospin, Germany) at 1 and 3 days after the induction of brain injury. The mice were anaesthetized with isoflurane (3% for induction and 1–1.5% for maintenance) and positioned on an animal cradle with a stereotaxic head holder. The respiration and temperatures of the mice were monitored continuously during the scanning process. To monitor brain oedema evolution, T2-weighted images were acquired at each imaging time point. The setup parameters were as follows: repetition time, 3000 ms; echo time, 30 ms; field of view, 30 × 30 mm^2^; image matrix, 256 × 256; slice thickness, 0.5 mm. Brain oedema changes were quantified by measuring the T2-hyperintense area using Weasis software. Each whole-brain scan was composed of 28 slices.

### Brain water content

After anaesthetization and euthanasia, the mice were decapitated at 24 and 72 h after TBI, and their brains were immediately removed and divided into three parts: the right hemisphere, left hemisphere, and cerebellum for experiment 1 and the whole cerebrum for experiments 3 and 4. Each brain region was weighed immediately to determine the wet weight and then dried for 24 h at 100 °C to obtain the dry weight. The percentage of brain water content was calculated as follows: (wet weight—dry weight)/wet weight × 100%. The percentage of water content was calculated by 2 trained investigators who were blinded to the animal grouping.

## Behavioural tests and neurological scoring

### Neurological deficit score

The acute neurologic deficits score was determined at 1, 3, 5, and 7 days (n = 10) after the administration of CYM-5442 using a 28-point scoring system as described previously [13, 27]. Body symmetry, gait, climbing, circling behaviour, front limb symmetry, compulsory circling, and whisker responses were assessed. All data were recorded by 2 observers blinded to the mouse grouping, and the average score of the subscales was used as the final score of each mouse.

### Hanging wire test

In this study, grip strength was assessed by placing mice on an apparatus consisting of a steel wire (50 cm long; 2 mm in diameter) pulled between two vertical supports and suspended 40 cm over a flat surface; a soft surface was placed below the wire to prevent physical trauma to the mice. The mice were placed on the wire midway with two forepaws and were observed for 60 s in 3 trials. The mice were evaluated as follows: 0, fell off; 1, hung onto the wire with 2 forepaws; 2, same as for 1 with an added attempt to climb onto the wire; 3, hung onto the wire with two forepaws and 1 or both hind paws; 4, hung onto the wire with forepaws and with tail wrapped around the wire; and 5, escaped to one of the platforms. The average score of three successive trials was taken for each animal. All tests were performed by two investigators blinded to the experimental groups.

### Beam-walking test

In the beam-walking test, the mice were placed at one end of a wooden beam (12 mm in diameter, 1 m long, and 50 cm high) and allowed to traverse the beam into a black box located at the end of the beam. The number of foot faults and the time to complete the task were documented. A foot fault was defined as any paw slipping off the top surface of the beam. Data were analysed by the primary experimenter and confirmed by a second experimenter via video recording. Before surgery, the mice were trained for 3 days, and the performance was measured on days 1, 3, 5, and 7 post-CCI. All experimenters and animal handlers were blinded to the treatment groups.

### Blood counts

At 3 h, 24 h, 72 h and 7 days after TBI, blood was collected from the retro-orbital venous plexus of anaesthetized mice into EDTA-coated capillary tubes from, and no drugs were administered on the day of blood collection except for from mice in the group at 3 h after TBI. Blood samples were analysed using an autoanalyzer (XT-2000i, Sysmex Corporation) to detect lymphocytes and leukocytes.

### BBB permeability assays

To measure BBB permeability, 2% Evans Blue (EB, 4 mL/kg) in sterile saline was injected through the tail vein 1 h before the animals were sacrificed. The mice were transcardially perfused with saline, and their brains were dissected and weighed. The samples were then homogenized in PBS (1 ml/300 g), sonicated for 2 min, and centrifuged at 15,000 rpm for 5 min at 4 °C, and the supernatant was then collected in aliquots. Next, 500 μL of 50% trichloroacetic acid was added to each 500 μL of supernatant and incubated overnight at 4 °C. Finally, these samples were centrifuged at 15,000 rpm for 30 min at 4 °C. The samples were detected with a spectrometer at 610 nm and quantified using a standard curve that was normalized to tissue weight (μg/g). Then, to assess the fluorescence intensity, the brains were removed in preparation for coronal brain sectioning. Red autofluorescence of EB was observed on the slides as previously described [28]**.** The mean red autofluorescence of EB was evaluated by 2 observers blinded to the mouse treatment groups.

### Immunofluorescence microscopy

After deep anaesthetization, the mice were perfused with PBS and 4% PFA. Their brains were removed and immersed in 4% paraformaldehyde, 20% sucrose, and 30% sucrose in sequence for fixation and dehydration, and the tissues were embedded in optimal cutting temperature (OCT; Sakura) compound. Coronal brain Sects. (20-µm thickness) were subjected to immunofluorescence (IF) staining. After washing with PBS and PBS + 0.4% Triton X-100, the brain sections were blocked with 10% goat serum for 1 h at 37 °C, incubated with primary antibodies overnight at 4 °C and washed three times with PBST. Then, the cryosections were incubated with secondary antibodies conjugated to Alexa Fluor 488/594/647 for 1 h at room temperature. The above protocol was repeated once for double staining. Endothelial marker CD31 was used as a loading control and for the normalization in quantification. The primary antibodies included anti-Mfsd2a (1:50, Invitrogen, USA, PA5-21049), anti-caveolin-1 (1:200, CST, USA, 3267S), anti-claudin-5 (1:100, Invitrogen, USA, 34-1600), anti-CD31 (1:50, Genetex, UK, 34-1600), and anti-albumin (1:200, Abcam, USA, ab207327). For each sample, eight Sects. (20-µm thickness) at intervals of 300 mm per mouse were performed immunostaining and analysed, random fields of view were selected within the area of interest, as well as the cross-sectional areas were measured and represented as the average of four independent measurements. All measurements were performed by 2 observers blinded to the mouse treatment factors. Images were captured by confocal microscopy (Zeiss, LSM780, Germany) and processed using ImageJ and Adobe Photoshop.

### Western blotting

Brain tissues were collected, and protein samples from pericontusional cortex tissues were extracted in RIPA lysis buffer (containing protease and phosphatase inhibitors) as described previously to perform Western blotting [11]. Equal amounts of protein samples (50 μg) were separated by SDS–PAGE and transferred onto polyvinylidene difluoride (PVDF) membranes. The PVDF membranes were blocked in 5% nonfat milk for 1 h at room temperature and then incubated overnight at 4 °C with the following primary antibodies: anti-Mfsd2a (1:1000, Invitrogen, USA, PA5-21049), anti-caveolin-1 (1:1000, CST, USA, 3267S), anti-claudin-5 (1:500, Invitrogen, USA, 34-1600), anti-albumin (1:1000, Abcam, USA, ab207327), and anti-GAPDH (1:1000, Proteintech, China, 10494-1-AP). The following day, the membranes were incubated with appropriate secondary antibodies for 1 h at room temperature. Immunoblots were visualized with a Fusion-FX7 system (Vilber Lourmat, Chongqing, China) and quantified with optical methods using ImageJ software. GAPDH was used as the loading control in each blot. All WB experiments were repeated 3–5 times.

### Infection with recombinant adeno-associated virus

The recombinant HBAAV2/9-CMV-m-Mfsd2a-3xflag-Null (Mfsd2a overexpression) and HBAAV2/9-ZsGreen control viruses were generated by Hanbio Technologies (Shanghai, China). For in vivo infection, an AAV overexpressing Mfsd2a (2 μl, 1.2*10^12^ viral genomes/mL) or a control virus (2 μl, 1.3*10^12^ viral genomes/mL) was administered via intracerebroventricular injection using a slow infusion rate of 100 nl/min. Similarly, the recombinant HBAAV2/9-m-Mfsd2a shRNA2-Null (downregulated Mfsd2a) and HBAAV2/9-mCherry NC control viruses were generated by Hanbio Technologies (Shanghai, China). For in vivo infection, an AAV with Mfsd2a knockdown (2 μl, 1.7*10^12^ viral genomes/mL) or a control virus (2 μl, 1.3*10^12^ viral genomes/mL) was administered via intracerebroventricular injection. TBI was induced 4 weeks after recovery from the intraventricular virus injection. The infectivity of the purified viruses was evaluated by fluorescence microscopy and WB (Additional file 1: Figure S1).

### S1P measurement by enzyme-linked immunosorbent assay (ELISA)

The S1P concentration was measured according to previous experiments [22]. Five milligrams of tissue was transferred into a silicified glass tube, cut into 1 mm^3^ pieces and incubated in 0.4 ml of PBS at 4 °C. After 20 min, the supernatant was separated from the tissue fragments, which were again homogenized in 0.4 ml (1:1) of methanol/water. The amount of S1P in the supernatant was measured by a mouse S1P ELISA kit (MEIKE Jiangsu Sumeike Biological Technology Co., Ltd) according to the manufacturer’s instructions. The amount of S1P was normalized to the protein content.

### Transmission electron microscopy

After anaesthetization, mice were transcardially perfused with ice-cold saline followed by 4% PFA and 2.5% glutaraldehyde with 0.1 mol/L PBS buffer. The pericontusional cortex tissues were microdissected into 1 mm^3^ specimens. Then, these samples were immersion-fixed in 2% glutaraldehyde for 24 h. After washing with PBS, they were fixed in 1% osmium tetroxide in 0.1 M PBS for 45 min. The specimens were dehydrated in increasing concentrations of acetone and embedded in Araldite resin. Ultrathin 60-nm-thick sections were cut using a diamond knife on a Leica UCT ultramicrotome (Diatome, Wetzlar, Germany). The prepared ultrathin sections were mounted on a copper grid, stained with uranium acetate and lead citrate, and observed with a JEM-1400Plus TEM (H-7500, Hitachi LTD., Japan). TEM imaging of horseradish peroxidase (HRP) injection was performed as previously described [12]. Mice were anaesthetized, and HRP type II (Sigma Aldrich, P8250-50KU, 0.5 mg/g body weight dissolved in 400 μL of PBS) was injected through the tail vein. The brain was collected after 30 min of circulation, and the brain tissue was soaked in a 0.1 m cacodylate-buffer mixture (5% glutaraldehyde and 4% PFA) for 1 h at room temperature, rinsed overnight with 4% PFA/0.1 m sodium cacodylate at 4 °C and cut into 50-µm-thick free-floating sections using a vibrotome. Sections were incubated in 3–3' diaminobenzidine (DAB, ZSGB-BIO, ZLI-9018, 5 mg/10 mL in 0.05 M Tris–HCl pH 7.6 buffer) with 0.01% hydrogen peroxide for 45 min at room temperature. After DAB staining, the sections were postfixed in 1% osmium tetroxide and 1.5% potassium ferrocyanide, dehydrated and embedded in epoxy resin. Ultrathin Sects. (80 nm) were then cut from the block surface, collected on copper grids, stained with Reynold’s lead citrate and examined under a Hitachi-7500 electron microscope. For each sample, four 1 mm^3^ specimens sections from pericontusional cortex tissues were performed TEM and twenty cortical vessels from four sections that were comparable in size were analysed for vesicle quantification, random fields of view were selected within the area of interest, as well as the cross-sectional areas were measured and represented as the average of four independent measurements. All measurements were performed by 2 observers blinded to the mouse treatment factors.

### Statistical analysis

All statistical analyses were performed with Prism 9 software (Prism, GraphPad, San Diego, USA). Data are expressed as the mean ± standard deviation (SD). The Pearson test and Shapiro–Wilk test were used to analyze the normality and homogeneity of the data. Differences between two groups were analysed by Student’s t test (two-tailed), and multiple group comparisons were made using a one-way ANOVA followed by a Tukey’s multiple comparisons test. Differences in means across groups with repeated measurements over time were analyzed using the repeated-measures ANOVA. When the ANOVA showed significant differences, pairwise comparisons between means were tested by post hoc Bonferroni tests. P < 0.05 indicates statistical significance, and ns indicates not significant.

## Results

### CYM-5442 treatment attenuated neurological deficits and reduced brain oedema following TBI

**Fig. 2:**
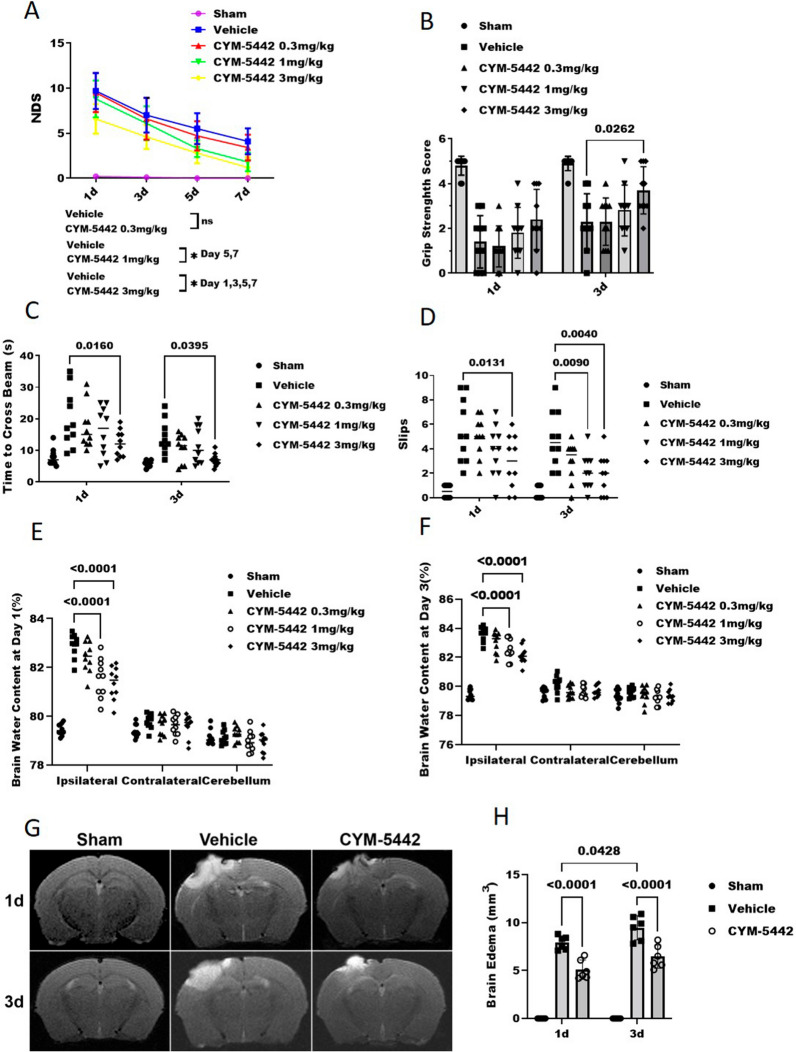
CYM-5442 treatment attenuated neurological deficits and reduced brain oedema after TBI. A battery of neurological tests were performed at days 1, 3, 5 and 7 (**A**) and at days 1, 3 (**B**, **C**, **D**) after TBI to comprehensively assess the motor, sensory, reflex, and balance functions in groups of TBI mice receiving vehicle or CYM-5442 (0.3, 1, and 3 mg/kg). **A**, **B** Neurological dysfunction in each group was assessed by the neurological deficit score (NDS) (**A**) and hanging wire test (**B**). Y-scale 0–15 represents the NDS score, and higher NDS score indicates more severe neurological impairment in mice (**A**). **A** repeated-measures ANOVA, *P < 0.05, n = 10/group. **B** two-way ANOVA and Tukey’s multiple comparisons test, n = 10/group. **C**, **D** Administration of CYM-5442 at 3 mg/kg after TBI reduced the time required to traverse a narrow beam (**C**) and decreased the number of slips (**D**), indicative of better motor function and coordination. Two-way ANOVA and Tukey’s multiple comparisons test, n = 10/group. **E**, **F** The brain water content in each group was detected after TBI on days 1 (**E**) and 3 (**F**), and the results indicated that 1 and 3 mg/kg CYM-5442 treatment attenuated brain oedema following TBI. Two-way ANOVA and Tukey’s multiple comparisons test, n = 10/group. **G**, **H** magnetic resonance imaging (MRI) was performed to evaluate brain oedema, and the results indicated that 3 mg/kg CYM-5442 reduced brain oedema at days 1 and 3 after TBI. Two-way ANOVA and Tukey’s multiple comparisons test, n = 6/group. Those experimental groups in receipt of the vehicle/CYM-5442 also were subjected to TBI. All values are presented as the mean ± SD

Three different dosages of CYM-5442 were tested to select the optimal dosage for attenuating TBI-induced brain injury. The neurological deficit scores in the CYM-5442 treatment group were significantly lower compared with the TBI + Vehicle groups when administrated with dosage in 3 mg/kg at 1 day (6.60 ± 1.64 versus 9.70 ± 2.00, p = 0.011), 3 days (4.60 ± 1.35 versus 7.00 ± 1.94, p = 0.0379), 5 days (2.80 ± 1.13 versus 5.50 ± 1.71, p = 0.0061), 7 days (1.2 ± 1.13 versus 4.10 ± 1.44, p = 0.0064) and 1 mg/kg at 5 days (3.30 ± 0.94 versus 5.50 ± 1.71, p = 0.0225), 7 days (1.80 ± 1.03 versus 4.10 ± 1.44, p = 0.0009) but not 0.3 mg/kg (p > 0.05, day 1,3,5,7) (Fig. 2A). Significant neurological deficits were also observed in the TBI + Vehicle group at 24 h compared with the Sham group as assessed by the hanging wire test (1.40 ± 1.17 versus 4.80 ± 0.42, p < 0.0001) (Fig. 2B), time to cross beam (20.10 ± 9.07 versus 7.80 ± 2.70 s, p < 0.0001) (Fig. 2C) and the slips test (5.50 ± 2.59 versus 0.50 ± 0.52, p < 0.0001) (Fig. 2D). Administration of CYM-5442 (3 mg/kg) significantly improved the neurological outcomes at 24 h after TBI when compared with those of the TBI + Vehicle group as assessed by the NDS (Fig. 2A), time to cross beam (12.20 ± 4.21 versus 20.10 ± 9.07 s, p = 0.016) (Fig. 2C) and the slips test (2.90 ± 2.18 versus 5.50 ± 2.59, p = 0.0131) (Fig. 2D). Although the mice treated with three concentrations of CYM-5442 still had more severe cerebral oedema at 1 and 3 days after TBI compared with sham group, CYM-5442(3 mg/kg and 1 mg/kg) treatment significantly reduced cerebral oedema compared to the TBI + Vehicle group. The brain water content in the ipsilateral cortex was significantly increased in the TBI + Vehicle group compared with the Sham group at 24 h after TBI (82.90 ± 0.48 versus 79.46 ± 0.23, p < 0.0001) and was significantly reduced after CYM-5442 administration in 3 mg/kg (82.90 ± 0.48% versus 79.46 ± 0.23%, p < 0.0001) and 1 mg/kg (81.56 ± 0.79% versus 79.46 ± 0.23%, p < 0.0001) but not 0.3 mg/kg (p > 0.05) (Fig. 2E). Twenty-four hours after TBI, MRI evaluations also revealed that treatment with CYM-5442 (3 mg/kg) tended to reduce brain oedema when compared with TBI + Vehicle group (5.10 ± 1.01 versus 7.90 ± 0.69 mm^3^, p < 0.0001) (Fig. 2G, H).

To further verify the treatment efficacy of CYM-5442 (3 mg/kg), neurobehavioral tests and brain water content evaluations were also performed at 72 h after TBI. Consistently, compared to the TBI + Vehicle group, administration of CYM-5442 (3 mg/kg) after TBI improved grip strength (3.70 ± 1.05 versus 2.30 ± 1.25, p < 0.0262) (Fig. 2B), reduced the time required to traverse a narrow beam (7.00 ± 2.00 versus 14.10 ± 5.28 s, p < 0.0395) (Fig. 2C) and decreased the number of slips (1.90 ± 1.66 versus 4.80 ± 2.30, p < 0.0040) (Fig. 2D). The brain water content in the ipsilateral cortex was significantly reduced in the CYM-5442 (3 mg/kg) group (82.14 ± 0.60% versus 79.43 ± 0.35%, p < 0.0001) (p < 0.05, Fig. 2F), which resulted in significantly decreased brain oedema compared with that in the TBI + Vehicle group as determined by MRI scanning at 72 h after TBI (6.45 ± 1.19 versus 9.46 ± 1.27 mm^3^, p < 0.0001) (Fig. 2G, H).

Our results showed that CYM-5442 treatment attenuated neurological deficits and reduced brain oedema following TBI, and CYM-5442 3 mg/kg was better than CYM-5442 at lower concentrations.

### CYM-5442 3 mg/kg reduced circulating lymphocytes, but did not cause sustained immunosuppression

**Fig. 3:**
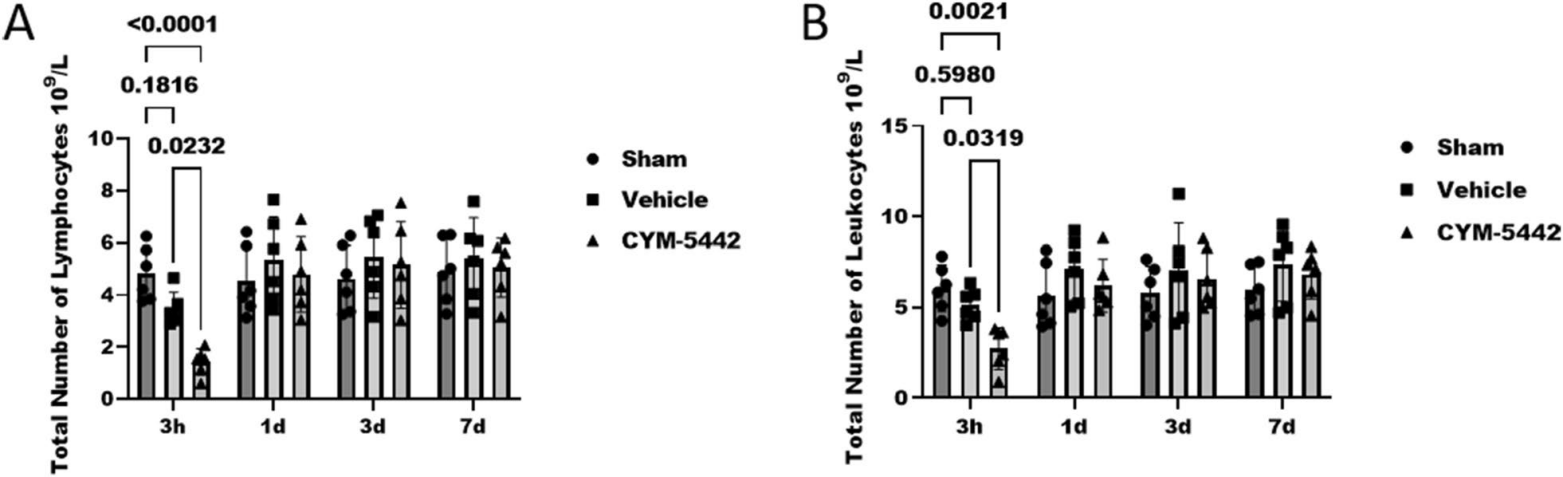
Treatment with CYM-5442 reduced circulating lymphocytes after TBI. **A** Total number of lymphocytes. Mice treated with 3 mg/kg CYM-5442 showed reduced lymphocyte numbers at 3 h after TBI. **B** Total number of leukocytes. Mice treated with 3 mg/kg CYM-5442 showed reduced leukocyte numbers at 3 h after TBI. The results showed no significant differences in the lymphocyte and leukocyte counts at days 1, 3, and 7 between the CYM-5442 (3 mg/kg) group and the Sham and TBI + Vehicle groups. Two-way ANOVA and Tukey’s multiple comparisons test were used for statistical analyses, n = 6/group. Those experimental groups in receipt of the vehicle/CYM-5442 also were subjected to TBI. All values are presented as the mean ± SD

Although the numbers of lymphocytes and leukocytes in peripheral blood were decreased at 3 h after TBI (Fig. 3A, B), the numbers of lymphocytes (3.47 ± 0.63 versus 1.43 ± 0.51 10^9^/L, p < 0.0232) and leukocytes (5.14 ± 0.88 versus 2.72 ± 1.15 10^9^/L, p < 0.0319) in TBI + Vehicle group mice were decreased significantly compared with CYM-5442 intervention group (Fig. 3A, B). To observe the effect of the continuous daily administration of CYM-5442 on blood counts, blood samples were collected at 24 h after the last drug administration. The lymphocyte counts at days 1, 3, and 7 in the CYM-5442 (3 mg/kg) group were not significantly different from those in the Sham and TBI + Vehicle groups (Fig. 3A, B). Our results confirmed that CYM-5442 transiently suppressed lymphocyte trafficking, but continuous daily use of CYM-5442 did not result in sustained immunosuppression.

Our results indicate that CYM-5442 3 mg/kg treatment attenuated neurological deficits and reduced brain oedema following TBI. Moreover, CYM-5442 3 mg/kg reduced circulating lymphocytes, but did not cause sustained immunosuppression. Taken together, based on its efficiency compared with the other two lower concentrations and its safety, 3 mg/kg CYM-5442 was selected for subsequent experiments.

### Treatment with CYM-5442 improved cerebral blood flow after TBI

**Fig. 4:**
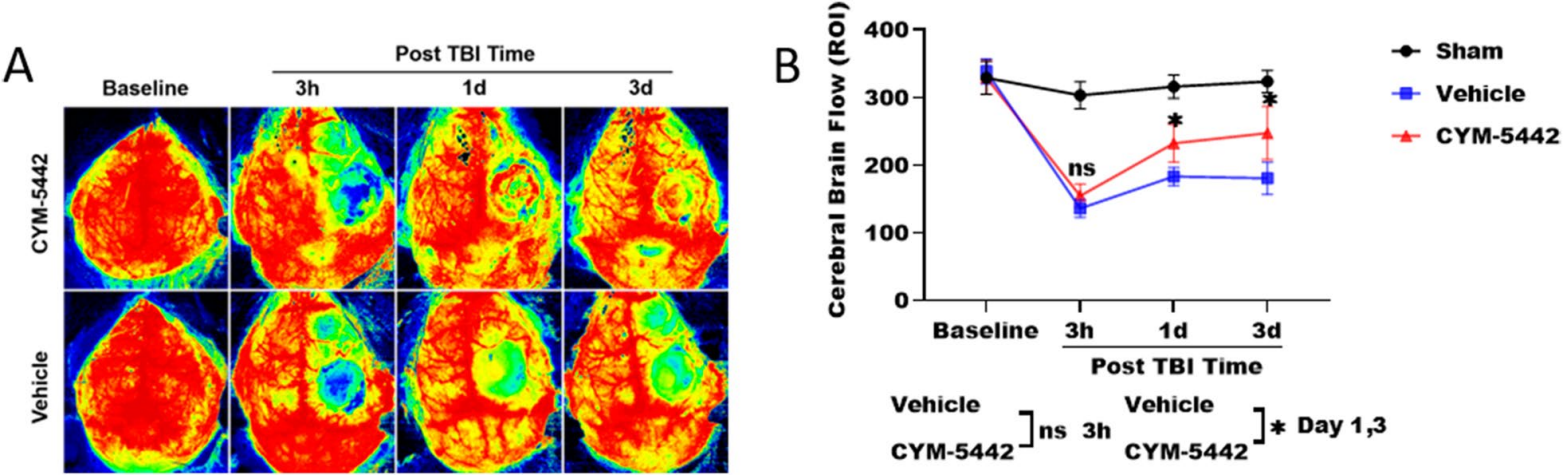
Treatment with CYM-5442 improved cerebral blood flow after TBI. **A** Laser speckle images of TBI mice treated with 3 mg/kg CYM-5442. **B** Blood flow of the contusional and pericontusional cortices in the regions of interest (ROIs) in TBI mice receiving 3 mg/kg CYM-5442 or Vehicle or Sham treatment. All data was analysed by repeated-measures ANOVA, followed by Tukey’s multiple comparisons test, *P < 0.05, n = 8. Those experimental groups in receipt of the vehicle/CYM-5442 also were subjected to TBI. All values are presented as the mean ± SD

In the acute phase of TBI, CBF is decreased significantly [10, 29]. To quantify the changes in local CBF after TBI, we explored the effect of CYM-5442 on cerebral perfusion using LCSI, a semiquantitative method. Consistent with our previous findings, perfusion in the lesion areas was obviously lower in the TBI + Vehicle group than in the Sham group at 3 h (136.07 ± 13.04 versus 303.36 ± 19.83, p < 0.0001), 1 day (183.58 ± 13.40 versus 316.13 ± 17.10, p < 0.0001), 3 days (180.76 ± 23.81 versus 323.79 ± 16.31, p < 0.0001) (Fig. 4A, B). In addition, CYM-5442 significantly improved CBF at 1 day (232.37 ± 28.34 versus 183.58 ± 13.40, p < 0.0035) and 3 days (247.66 ± 39.29 versus 180.76 ± 23.81, p < 0.0041) postinjury compared to that in the TBI + Vehicle group (Fig. 4A, B).

### Administration of CYM-5442 decreased BBB permeability and vesicle transcytosis after TBI

Recent studies have shown that CYM-5442 maintains BBB integrity in ischaemic stroke by restricting vesicle transcytosis, which underlies BBB dysfunction in the acute phase [24]. Disruption of the BBB and subsequent oedema formation can lead to worsening of neurological symptoms in patients with TBI [30]. For this reason, we analysed the consequences of CYM-5442 on BBB permeability following TBI. The extent of BBB damage was assessed by the determined concentration of the vascular tracer EB/albumin, which crosses the BBB primarily by transcellular transport [9].

**Fig. 5:**
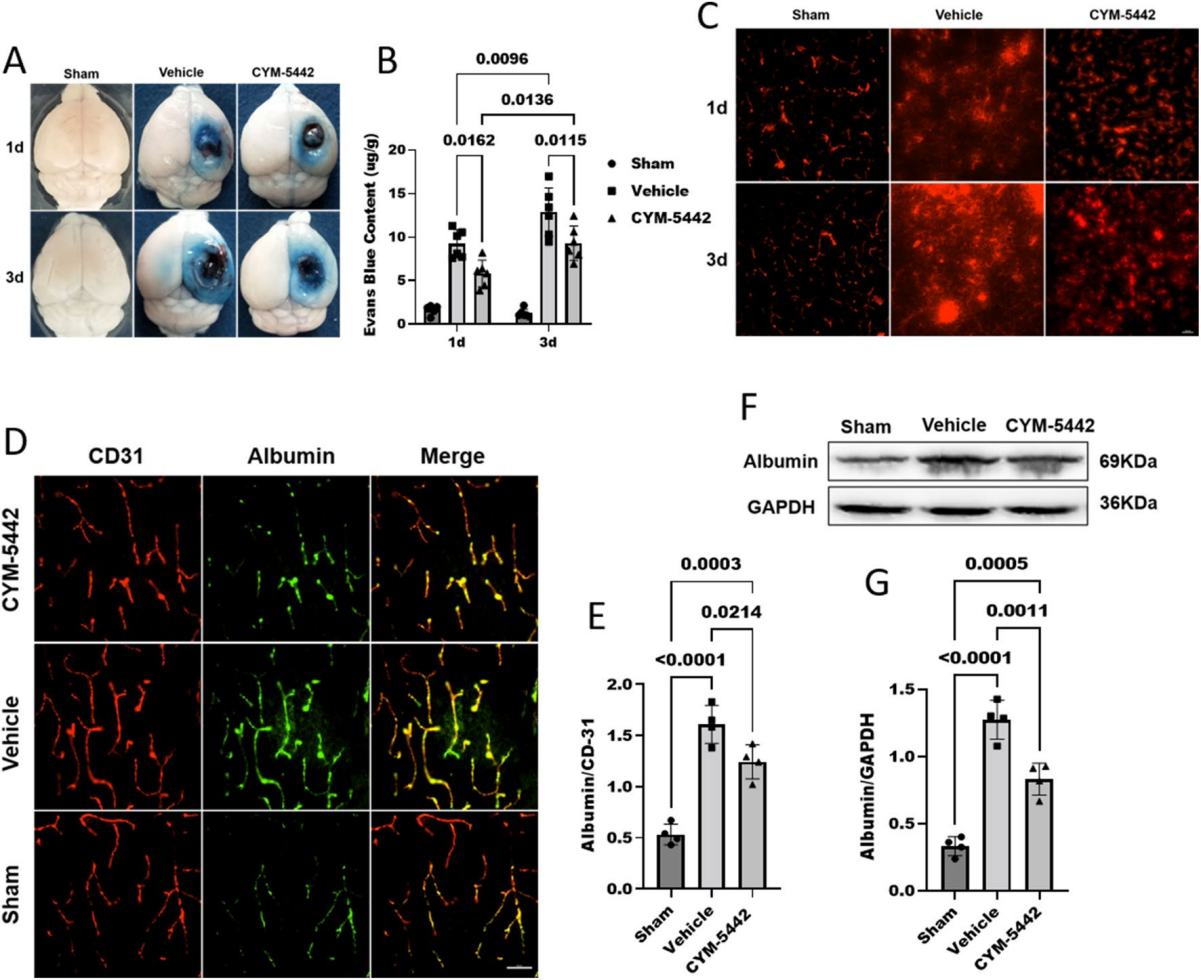
Administration of CYM-5442 decreased BBB permeability after TBI. **A**, **B**, **C** Representative images (**A**) and quantitative analyses (**B**) of the degree of EB dye extravasation in the ipsilateral cortices of mice in each group on days 1 and 3 after TBI. Two-way ANOVA and Tukey’s multiple comparisons test were used for statistical analyses, n = 6/group. Administration of CYM-5442 decreased the perivascular red EB fluorescence (Em: 680 nm) leakage from vessels of the ipsilateral cortex (**C**). Scale bar = 100 μm. **D**, **E** Immunostaining for albumin (green) and CD31-positive endothelial capillaries (green) in brain slices (**D**) and quantified (**E**). Endothelial marker CD31 was used as a loading control and for the normalization. Scale bar = 50 μm. One-way ANOVA, followed by Tukey’s multiple comparisons test, n = 4/group. **F, G** The levels of albumin in the brain tissues of mice from each group were detected by WB (F) and quantified (G) on day 3 after TBI. One-way ANOVA, followed by Tukey’s multiple comparisons test, n = 4/group. TBI was associated with increased EB dye extravasation and albumin leakage compared to that in the Sham group. Administration of CYM-5442 significantly decreased the EB dye extravasation as well as albumin leakage dye extravasation compared to that in nontreated TBI mice. Those experimental groups in receipt of the vehicle/CYM-5442 also were subjected to TBI. All values are presented as the mean ± SD

We confirmed that CYM-5442 improved BBB function by evaluating EB dye extravasation with spectrometric and quantitative perivascular EB fluorescence analyses. EB dye leakage was significantly increased in border (Fig. 5A) and depth (Additional file 2: Figure S2) from the pericontusional cortical surface of the TBI + Vehicle group compared to the Sham group. However, this increase in EB extravasation was significantly attenuated by CYM-5442 treatment at 1d and 3d post-TBI. EB fluorescence was largely confined to the vasculature of the sham group mice and was easily detected in pericontusional cortical extravascular spaces. Administration of CYM-5442 decreased the perivascular red EB fluorescence (Em: 680 nm) leakage from vessels of the ipsilateral cortex (Fig. 5C). Quantification of perivascular EB fluorescence revealed that administration of CYM-5442 prevented BBB destruction and decreased EB extravasation from the vasculature compared with the TBI + Vehicle group at day 1 (5.81 ± 1.54 versus 9.24 ± 1.61, p < 0.0162) and day 3 (9.31 ± 1.98 versus 12.87 ± 2.78, p < 0.0115). (Fig. 5A–C).

We then further determined the effects of CYM-5442 on BBB permeability at 72 h after TBI by evaluating albumin leakage by IF and WB analyses. Mice subjected to TBI showed significantly increased albumin leakage in the pericontusional cortex compared to that of Sham mice at 72 h after TBI, and CYM-5442 remarkably reduced the albumin leakage compared to that in the TBI + Vehicle group (1.24 ± 0.16 versus 1.61 ± 0.18, p < 0.0214) (Fig. 5 D, E). WB results revealed that the expression of albumin was significantly increased after TBI, and CYM-5442 reversed the TBI-induced increases in albumin levels, further confirming the protective effect of CYM-5442 on BBB functions following TBI (Fig. 5F, G).

### Cerebrovascular endothelial cells contained high levels of transcytosis vesicles after TBI

**Fig. 6:**
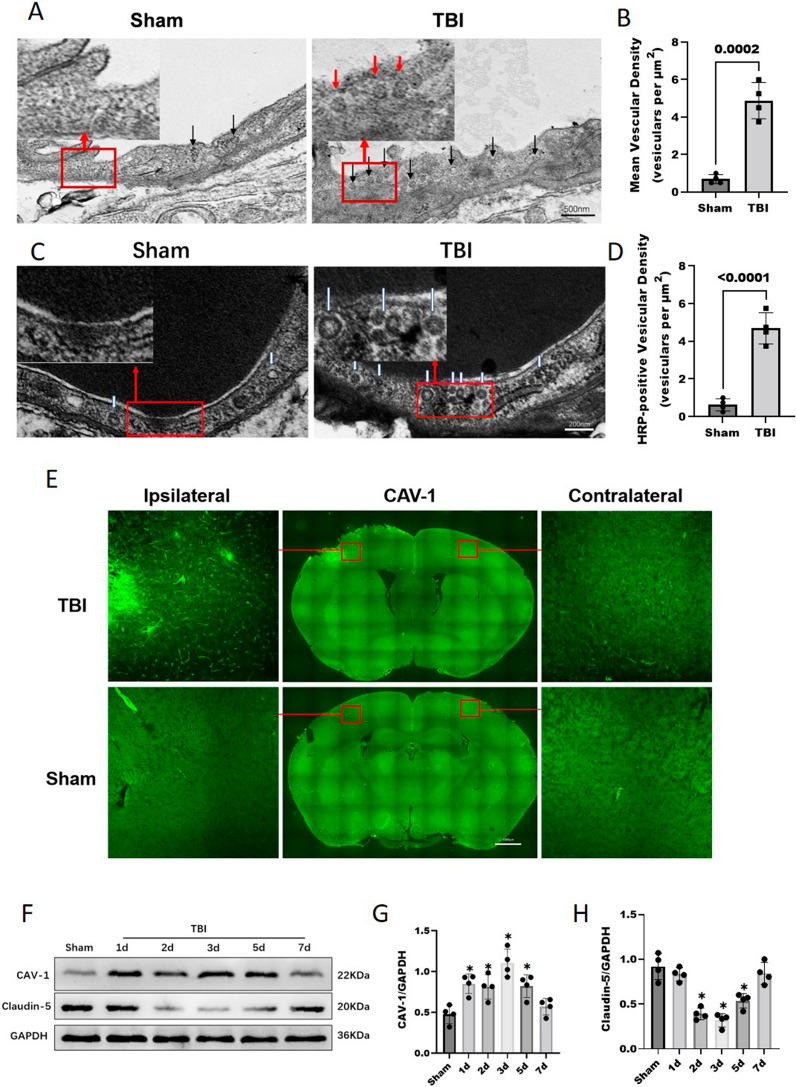
Cerebrovascular ECs contain high levels of transcytotic vesicles after TBI. **A** TEM images show more transcytosis vesicles (black arrows) in ECs (ECs) after TBI compared with their controls. Scale bar = 500 nm. **B** Quantification of the transcytosis vesicles densities in mice of the TBI and Sham groups. Data was analyzed by two-tailed student t-test, n = 4/group. **C**, **D** HRP was injected into the tail veins of mice after TBI and into those of the corresponding controls, followed by TEM. An electron-dense DAB reaction (black) was observed in the blood vessel lumens of HRP-injected mice. HRP-filled vesicles (white arrowheads) (**C**) were observed in ECs and quantified (**D**). Data was analyzed by two-tailed student t-test, n = 4/group. Scale bar = 200 nm. **E** Representative immunofluorescence images of CAV-1 protein expression on day 3 after TBI. Each red square represents the respective magnified inset. The expression of CAV-1 was significantly increased after TBI. Bar = 1000 μm. **F**, **G**, **H** Representative WB analysis and quantification of the relative densities of the CAV-1 (**G**) and claudin-5 (**H**) bands. Data was analyzed by one-way ANOVA and Tukey’s multiple comparisons test, *P < 0.05 compared with the Sham group, n = 4/group. All values are presented as the mean ± SD

To evaluate whether TBI affects EC transcytosis, the transcytosis vesicles of cerebrovascular ECs were observed by TEM after TBI. Compared with that in the Sham group, the number of transcytosis vesicles in vascular ECs of the pericontusional cortex tissues was significantly increased in TBI mice (4.87 ± 0.96 versus 0.68 ± 0.23, p = 0.0002) (Fig. 6A, B), indicating more extensive cerebrovascular EC endocytosis. To further investigate whether the observed EC vesicles could functionally transport circulating substances, mice were injected with HRP through the tail vein for further observation by TEM. The number of HRP-positive vesicles in TBI group mice was significantly increased compared to that in the Sham group (4.68 ± 0.82 versus 0.62 ± 0.32, p < 0.0001) (Fig. 6C, D), further supporting the observed increased functional EC transcytosis after TBI.

Finally, we examined the expression level of caveolin-1 (CAV-1), a major component and marker of caveolar vesicles [31]. IF staining revealed that the expression of CAV-1 was significantly increased after TBI (Fig. 6E), and the WB results showed that compared with those in the Sham group, the CAV-1 protein levels in the TBI group were significantly increased at day 1 (0.83 ± 0.12 versus 0.47 ± 0.11, p = 0.0040), day 2 (0.81 ± 0.14 versus 0.47 ± 0.11, p = 0.0097), day 3 (1.10 ± 0.16 versus 0.47 ± 0.11, p < 0.0001) and day 5 (0.82 ± 0.13 versus 0.47 ± 0.11, p = 0.0078) after TBI and recovered on day 7 (0.56 ± 0.10 versus 0.47 ± 0.11, p = 0.7807) (Fig. 6F, G). Moreover, the WB results showed that the claudin-5 protein expression levels were decreased significantly at day 2 (0.39 ± 0.06 versus 0.91 ± 0.14, p < 0.0001), 3 (0.31 ± 0.07 versus 0.91 ± 0.14, p = p < 0.0001) and 5 (0.53 ± 0.08 versus 0.91 ± 0.14, p = 0.0002) after TBI (Fig. 6F, H), suggesting that in addition to damage to the TJ complex, vesicle transport was increased significantly after TBI.

### Mfsd2a protein expression was decreased after TBI, which resulted in a decreased S1P concentration in the brain parenchyma

**Fig. 7:**
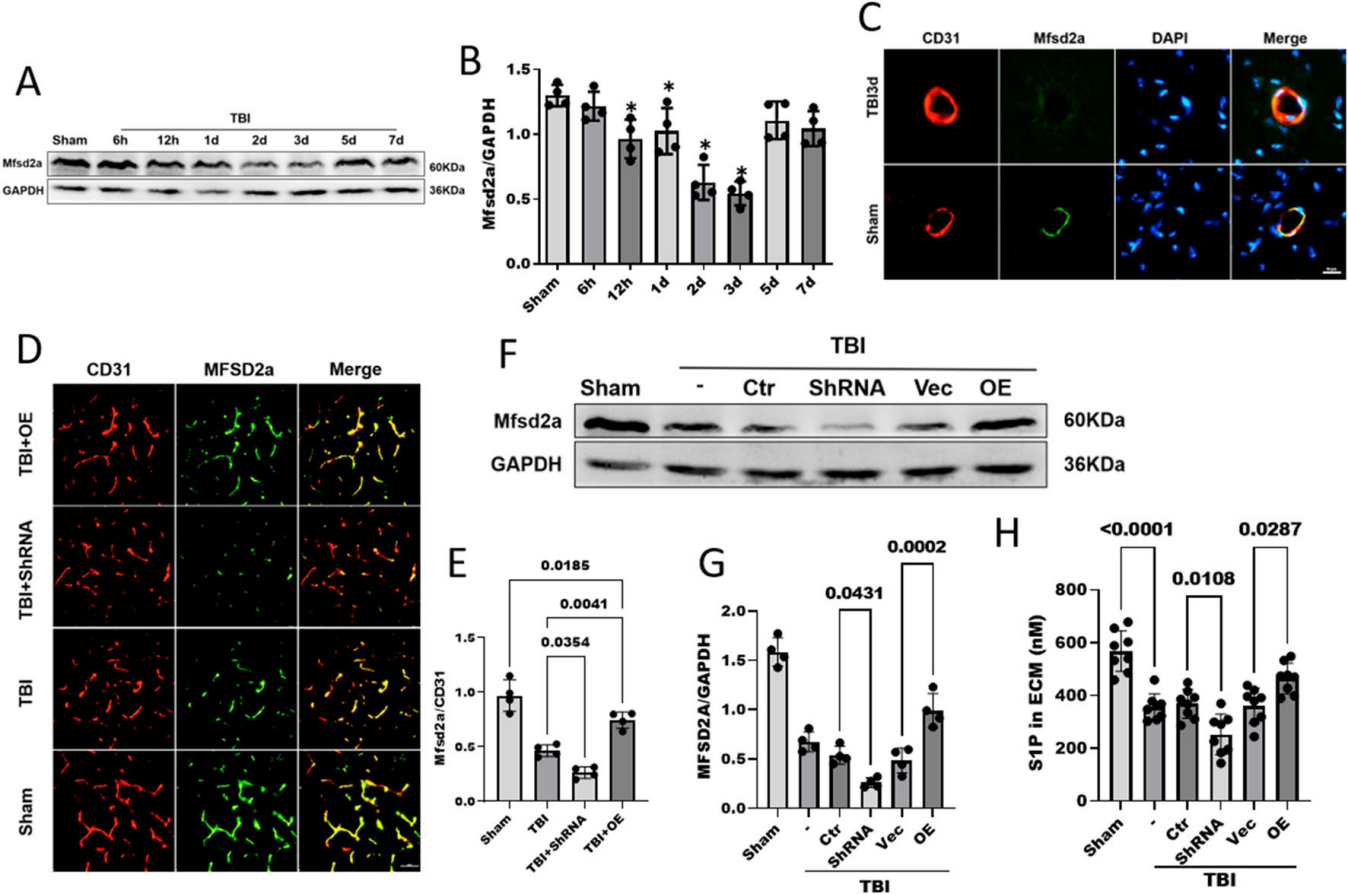
Mfsd2a protein expression was decreased after TBI, and Mfsd2a deficiency resulted in a decreased concentration of S1P in the brain parenchyma. **A**, **B** Mfsd2a protein expression at different time points after TBI as measured by Western blotting. n = 4/group. **C** Immunofluorescence staining was performed with antibodies for Mfsd2a (green) and CD31 (red), and nuclei were stained with DAPI (blue). Scale bar = 10 μm. **D**, **E** Immunofluorescence staining revealed differential Mfsd2a protein expression (red) in brain ECs (green) (**D**). The corresponding statistics are shown in (**E**). n = 4/group. Scale bar = 50 μm. **F**, **G** WB analyses of the protein levels of Mfsd2a in brain tissues on day 3 after TBI. n = 4/group. **H** Concentrations of total S1P in the brain ECM on day 3 after TBI. n = 8/group. All data was analyzed by one-way ANOVA and Tukey’s multiple comparisons test, *P < 0.05 compared with the Sham group. All values are presented as the mean ± SD. (-): TBI only; (Ctr): control shRNA; (Vec): vehicle; (OE): Mfsd2a overexpression

Cerebrovascular ECs contain high levels of endocytic vesicles after TBI, and Mfsd2a is known to negatively regulate endocytosis [32]. We next assessed whether the effect of TBI on EC endocytosis was mediated by Mfsd2a. WB and IF staining were used to detect the protein expression of Mfsd2a in pericontusional brain tissues. The WB results showed that the Mfsd2a protein levels in the pericontusional tissues were significantly decreased at 12 h (0.96 ± 0.14 versus 1.29 ± 0.08, p = 0.0086), 24 h (1.02 ± 0.17 versus 1.29 ± 0.08, p = 0.0386), 48 h (0.62 ± 0.13 versus 1.29 ± 0.08, p < 0.0001) and 72 h (0.54 ± 0.09 versus 1.29 ± 0.08, p < 0.0001) following TBI compared with those in the Sham group (Fig. 7A, B). The protein expression of Mfsd2a reached the lowest level 3 days after TBI and then gradually increased. Double IF staining showed that colocalization of Mfsd2a with CD31-positive ECs in group at 72 h after TBI decreased in the cerebrovasculature compared with that in the Sham group (Fig. 7C). These results suggest that the reduction in Mfsd2a expression after TBI may contribute to BBB disruption resulting from enhanced transcellular vesicle transport after TBI.

The S1P concentration directly affects the maintenance of the BBB. Although S1P is exported by Spns2 in brain ECs, Mfsd2a improves the efficiency of S1P transport [22]. Since CYM-5442, as an agonist of S1P1, reduces post-TBI vesicle transport, we explored whether Mfsd2a affects changes in the S1P concentration after TBI. Mfsd2a was suppressed by the injection of the HBAAV2/9-M- Mfsd2a -SHRNA2-NULL virus and overexpressed by the injection of the HBaAV2/9-CMV-M-Mfsd2a-3xFlag-NULL virus. WB analysis verified the Mfsd2a protein levels after the ICV administration of the AAVs (Figure. S1). Next, we detected the Mfsd2a protein levels in each group after TBI by IF and WB. IF staining showed characteristic expression of Mfsd2a in cerebrovascular ECs. After TBI, the expression level of Mfsd2a was decreased and was correspondingly upregulated (0.74 ± 0.07 versus 0.46 ± 0.05, p = 0.0041) and downregulated (0.26 ± 0.05 versus 0.46 ± 0.05, p = 0.0354) by the viruses compared with the TBI + Vehicle group (Fig. 7D, E). Consistent with this finding, the WB results also showed that the shRNA-mediated knockdown of Mfsd2a significantly reduced the expression of Mfsd2a after TBI compared that in the control group (0.26 ± 0.04 versus 0.53 ± 0.09, p = 0.0431), and similarly, AAV overexpression of Mfsd2a increased the expression of Mfsd2a after TBI (0.99 ± 0.17 versus 0.48 ± 0.12, p = 0.0002) (Fig. 7F, G).

To determine whether Mfsd2a affects S1P export in the brain, we subsequently measured the concentration of S1P in the extracellular matrix (ECM). The ELISA data showed that the S1P concentration in the brain ECM was significantly decreased after TBI (568.16 ± 77.34 versus 356.13 ± 50.21 nM, p < 0.0001). The reduction in Mfsd2a expression induced by AAV further reduced the S1P concentration in the ECM (252.48 ± 76.62 versus 368.13 ± 54.56 nM, p = 0.0108), while the overexpression of Mfds2a reversed the decrease in S1P (464.85 ± 57.62 versus 360.94 ± 66.86 nM, p = 0.0287) (Fig. 7H). We demonstrated that TBI decreases the expression of Mfsd2a, which reduces the concentration of S1P in the ECM, and this pathological change may be involved in increased vesicle transport after TBI.

### Reduced Mfsd2a expression after TBI resulted in increased CAV-1-mediated vesicle transport and BBB disruption

**Fig. 8:**
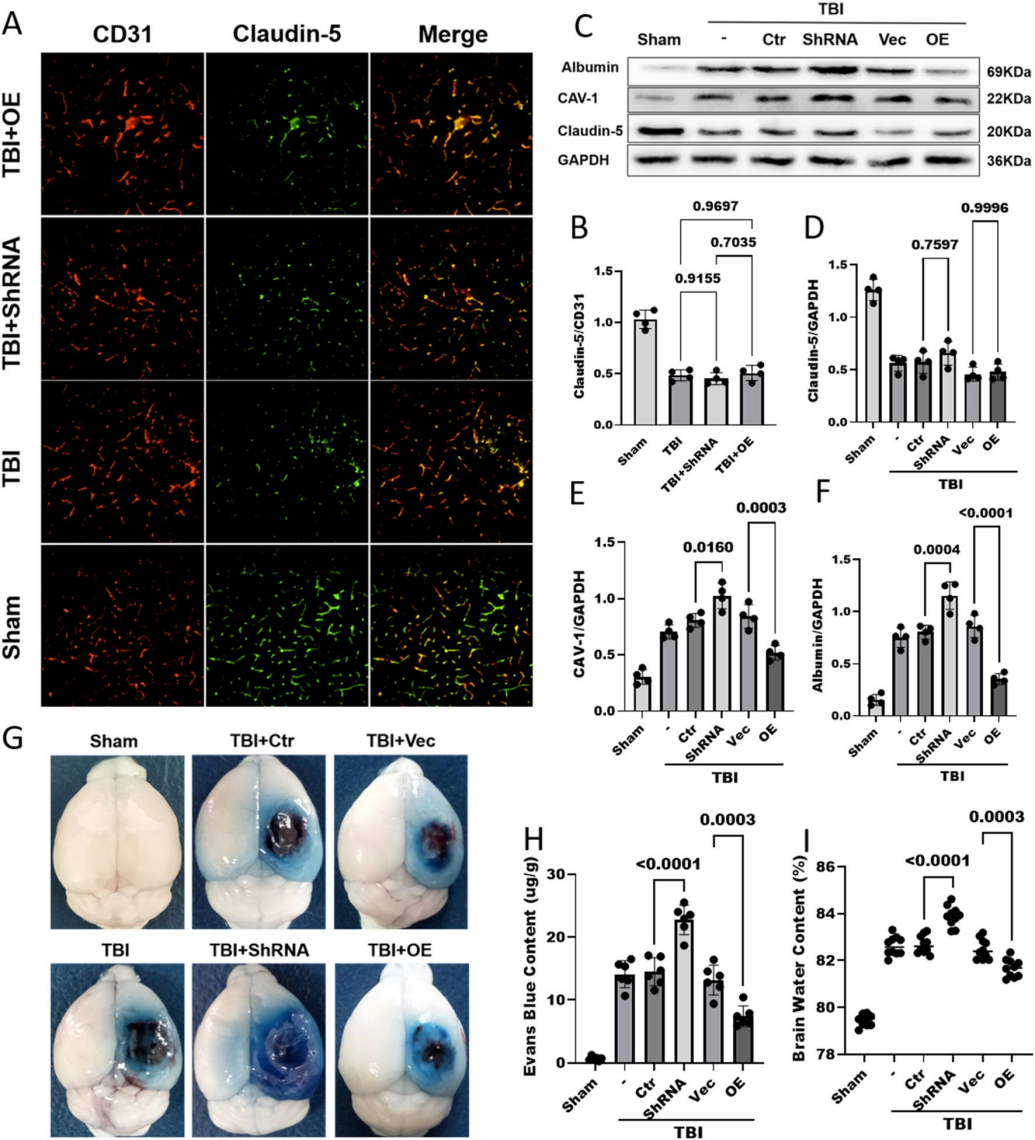
In addition to TJ disruption leading to BBB damage, decreased Mfsd2a expression after TBI leads to an increase in CAV-1-mediated vesicle transport and is involved in BBB damage. **A**, **B** Immunostaining for claudin-5 (green) and CD31-positive endothelial capillary profiles (red) in brain slices; nuclei were stained with DAPI. scale bar = 100 μm (**A**). The corresponding statistics are shown in (**B**). n = 4/group. **C**, **D**, **E**, **F** The protein levels of claudin-5 (**D**), caveolin-1 (**E**) and albumin (**F**) in pericontusional brain tissues on day 3 after TBI were measured by WB. n = 4/group. **G**, **H** Representative images (**G**) and quantitative analyses (**H**) of the degree of EB dye extravasation in the ipsilateral cortices of each group on day 3 after TBI. n = 6/group. **I** The brain water content in each group on day 3 after TBI. n = 10/group. All data was analyzed by one-way ANOVA and Tukey’s multiple comparisons test, all values are presented as the mean ± SD. (-): TBI only; (Ctr): control shRNA; (Vec): vehicle; (OE): overexpression of Mfsd2a

After TBI, Mfsd2a was downregulated, resulting in a decreased S1P concentration in the brain parenchyma. Since S1P is involved in BBB formation and functional maintenance and Mfsd2a negatively regulates CAV-1-mediated vesicle transcytosis, we observed changes in the BBB after TBI by upregulating and downregulating Mfsd2a expression. We evaluated the changes in TJs after TBI by detecting the expression level of Claudin-5 by IF and WB. Both methods showed that compared with that in the Sham operation group, the expression of Claudin-5 in all groups subjected to TBI was significantly decreased. However, the expression of Claudin-5 did not significantly differ between groups with Mfsd2a upregulation and downregulation (Fig. 8A–D).

In addition to the disruption of TJs, CAV-1-mediated vesicle transcytosis plays an important role in BBB damage. WB was used to detect the level of CAV-1 in mouse brain tissue, revealing significantly lower levels in the Sham operation group than in all the TBI groups. After TBI, the CAV-1 content in the brains of mice with Mfsd2a downregulation was significantly increased (1.02 ± 0.11 versus 0.80 ± 0.06, p = 0.0160), while that in the brains of mice with Mfsd2a overexpression was significantly decreased (0.51 ± 0.06 versus 0.84 ± 0.10, p = 0.0003) (Fig. 8C, E). Similar results regarding the brain albumin content were obtained by WB. Downregulation of Mfds2a expression after TBI further increased the brain level of albumin (1.15 ± 0.13 versus 0.80 ± 0.07, p = 0.0004), while upregulation of Mfsd2a expression reduced the increase in the brain albumin level after TBI (0.35 ± 0.05 versus 0.85 ± 0.10, p < 0.0001) (Fig. 8C, F). Similarly, the EB content in the Mfsd2a-ShRNA group was significantly higher than that in the control group (22.74 ± 2.35 versus 14.49 ± 2.22, p < 0.0001), while the EB extravasation in the overexpression group was lower than that in the control group (7.42 ± 1.64 versus 13.19 ± 2.37, p = 0.0003) (Fig. 8G, H). The brain water content in the group with Mfsd2a downregulation was significantly higher than that in the control group (83.89 ± 0.43% versus 82.69 ± 0.37%, p < 0.0001), while the brain water content after TBI was significantly reduced in the overexpression group (81.68 ± 0.38% versus 82.49 ± 0.43%, p = 0.0003) (Fig. 8I).

In the above experiments, downregulation of Mfsd2a expression after TBI aggravated BBB damage, while upregulation of Mfsd2a reduced BBB damage after TBI. Moreover, no significant differences were observed among the TBI group, control shRNA group, and Vehicle group. These results suggest that the downregulation of Mfsd2a expression after TBI leads to an increase in CAV-1-mediated vesicle transcytosis, which damages the BBB and exacerbates cerebral oedema.

### The S1P1 agonist CYM-5442 protects the BBB and alleviates cerebral oedema by reversing the increased vesicle transcytosis caused by the downregulation of Mfsd2a expression during TBI

CYM-5442 reduces the leakage of macromolecules (albumin/EB) from the BBB after TBI, suggesting that CYM-5442 reduce the vesicle transcytosis of vascular ECs. After TBI, the decreased expression of Mfsd2a led to a decrease in the ECM S1P concentration and an increase in vesicle transport, resulting in significant destruction of the BBB. We administered CYM-5442 after TBI to determine whether CYM-5442 could reverse the enhanced vesicle transport induced by the downregulation of Mfsd2a expression after TBI.

**Fig. 9:**
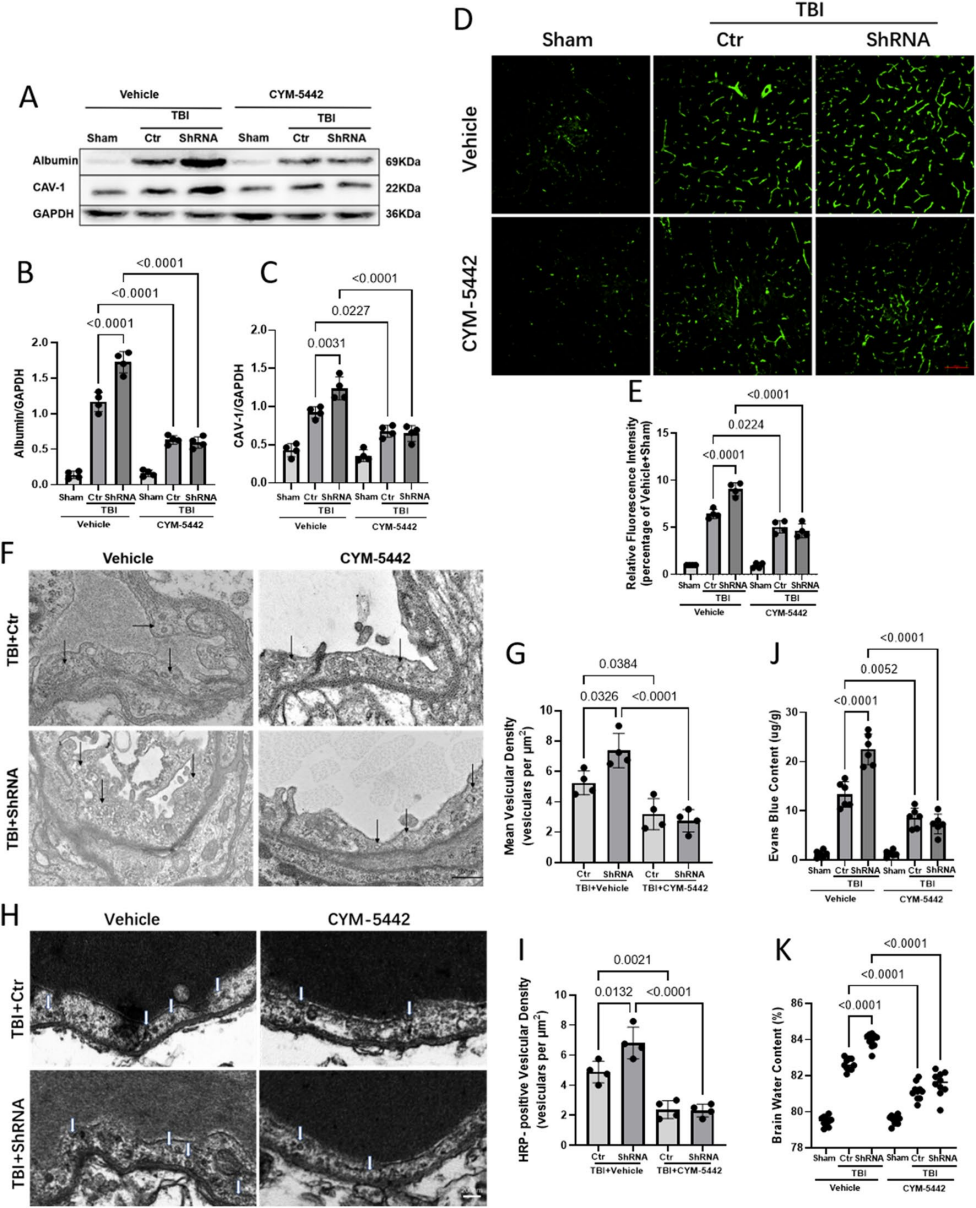
CYM-5442 protects against BBB disruption after TBI by reducing vesicle transcytosis. **A**, **B**, **C** The protein levels of albumin (**B**) and caveolin-1 (**C**) as measured by WB. n = 4/group. **D**, **E** Representative immunofluorescence images of CAV-1 protein expression. n = 4/group. Bar = 100 μm. **F**, **G** TEM images of transcytosis vesicles (black arrows) in ECs (ECs) after TBI. Scale bar = 500 nm (**F**). Quantification of the transcytosis vesicular density (**G**). n = 4/group. **H**, **I** HRP-filled vesicles (white arrowheads) (**H**) were observed in ECs and quantified (**I**). n = 4/group. Scale bar = 200 nm. **J** Quantitative analyses of the degree of EB dye extravasation in the ipsilateral cortices of each group on day 3 after TBI. n = 6/group. **K** Brain water content in the whole cerebrum in each group on day 3 after TBI. n = 10/group. All data was analyzed by one-way ANOVA and Tukey’s multiple comparisons test, all values are presented as the mean ± SD. (-): TBI only; (Ctr): control shRNA; (Vec): vehicle; (OE): overexpression of Mfsd2a

WB showed that the expression of albumin was significantly increased after TBI and further increased after Mfsd2a downregulation. CYM-5442 intervention had no significant effect on albumin expression in mice undergoing the Sham operation, while CYM-5442 intervention after TBI significantly reduced the albumin expression to the same level regardless of whether Mfsd2a was downregulated (0.63 ± 0.05 versus 0.59 ± 0.08, p = 0.9939) (Fig. 9A, B). We then examined the expression levels of CAV-1 and obtained results similar to those in the albumin study (Fig. 9A, C). Consistent with the WB results, IF staining revealed that the expression of CAV-1 was significantly enhanced at day 3 after TBI based on the fluorescence intensities in each group compared with that in the Vehicle + Sham group, and CYM-5442 reversed the increase in CAV-1 caused by the downregulation of Mfsd2a expression after TBI (4.64 ± 0.73 versus 9.05 ± 0.70, p < 0.0001) (Fig. 9D, E).

To observe the effect of CYM-5442 on transcytosis vesicle transport, TEM analysis was performed, showing that downregulation of Mfsd2a after TBI significantly increased vesicle transport (7.37 ± 1.12 versus 5.25 ± 0.79, p = 0.0326), while CYM-5442 significantly decreased vesicle transport in vascular ECs after TBI (3.18 ± 1.02 versus 5.25 ± 0.79, p = 0.0384) and reversed the increase in vesicle transport caused by the downregulation of Mfsd2a (2.75 ± 0.73 versus 7.37 ± 1.12, p < 0.0001) (Fig. 9F, G). TEM examination after the tail vein injection of HRP showed that CYM-5442 significantly reversed the increase in the number of HRP-positive vesicles caused by Mfsd2a downregulation after TBI (2.31 ± 0.42 versus 6.81 ± 1.04, p < 0.0001), which further confirmed that CYM-5442 compensated for the enhanced vesicle transport caused by Mfsd2a downregulation after TBI (Fig. 9H, I). In addition, CYM-5442 reduced the vesicular endocytosis caused by Mfsd2a downregulation after TBI, reduced cerebrovascular EB leakage after TBI (Fig. 9J), and significantly reduced cerebral oedema (Fig. 9K).

## Discussion

The present study demonstrated that treatment with CYM-5442 attenuates cerebral oedema, mitigates neurological deficits, and improves cerebral cortical blood flow in TBI mice. CYM-5442 can reduce peripheral blood lymphocytes and enhance the integrity of the BBB by reducing the vesicle transport of cerebrovascular ECs, which is sufficient to provide protection after TBI.

TBI is linked to several pathologies. Following TBI (primary mechanical injury), secondary brain damage is accompanied by various biomolecular and pathophysiological responses. Since these secondary events occur after the trauma and may appear within a few hours to days, fair chances can be availed for therapeutic targeting to prevent further deterioration. TBI is a complex, heterogeneous, and mechanobiological problem involving dynamic changes in the microenvironment following BBB disruption [33]. Multiple secondary pathological processes occur after TBI, and BBB breakdown is considered to be one of the initial changes [34]. The BBB consists of specific brain capillary ECs that are utilized for a highly specialized transport system [35], and the BBB regulates ion balance and nutrient transport and blocks the possibly detrimental particles that act as an interface between the CNS and peripheral circulatory system [36]. Disruption of the structure and function of the BBB disturbs neuronal functions and leads to neurological disruption, such as CBF alteration, water imbalance, cerebral metabolism imbalance, and the accumulation of inflammatory molecules [34]. Neuroinflammation exerts a negative effect on brain cells and plays a role in brain oedema [37].

BBB stability is maintained through two unique properties of vascular ECs, the presence of TJ proteins (TJPs) and a very low transcytosis rate [12]. Thus, enhanced BBB permeability under pathological brain conditions has been attributed to increased transcytosis [12, 13] or TJP degradation [38, 39]. Previous studies have found that increased transcytosis precedes TJP breakdown during stroke [12], suggesting that the upregulation of transcytosis is an early event in BBB breakdown occurring during these pathophysiological processes. However, most previously published studies have focused on only the BBB disruption caused by TJ changes after TBI [10, 11]. In contrast, whether transcellular transport also changes after TBI has not been elucidated. Similar to the pathological results of BBB destruction after stroke, in this study, we confirmed that transcellular and paracellular pathways accounts for changes in endothelial barrier properties during TBI progression, and increased caveolin-1 expression precedes decreased expression of claudin-5 during BBB breakdown after TBI. Mfsd2a is a transmembrane protein that is essential for the development and maintenance of the intact BBB and is the only well-identified molecule known to inhibit intracerebral EC caveolae-mediated endocytosis [40]. Under pathological conditions, Mfsd2a expression is decreased following brain injury in conjunction with the upregulation of caveolin-1 and BBB disruption. However, overexpression of Mfsd2a after brain injury can alleviate BBB disruption caused by increased vesicle transport. Therefore, Mfsd2a protects the BBB by reducing vesicular transcytosis [13–15]. As indicated above, our experiment also found that Mfsd2a expression was downregulated and that CAV-1-mediated vesicle transport was increased during the acute phase of TBI. Our study suggests that Mfsd2a downregulation plays a role in the increased BBB permeability after TBI, while Mfsd2a upregulation protects the BBB after TBI by inhibiting the transcytosis of ECs. Although Mfsd2a is a critical switch in the BBB, little is known about the mechanisms that regulate transcytosis at the BBB. Under physiological conditions, Mfsd2a transports docosahexaenoic acid (DHA) across the plasma membrane of brain ECs, forming a unique lipid composition that in turn suppresses caveolae formation to ensure BBB integrity [40, 41]. Mfsd2a has also been shown to regulate BBB formation and maintenance by interacting with SPNS2 to export S1P across brain ECs, suggesting that DHA is not the only factor regulating the BBB [22]. Mfsd2a deficiency causes insufficient transport of S1P in the brain parenchyma and then causes BBB breakdown by affecting S1P1[22]. Recent studies have also shown that S1P1 most likely maintains BBB integrity by restricting vesicle transport during ischaemic stroke, which underlies BBB dysfunction in the acute phase [24]. In conclusion, the decrease in the expression of Mfsd2a after TBI leads to a decrease in the ECM S1P concentration, which can directly affect BBB vesicle transport.

The bioactive sphingolipid S1P regulates numerous physiological processes, including lymphocyte trafficking, cardiac function, vascular development, and inflammation, through 5 G protein-coupled S1P receptors [42–45]. Its effect on immune cell trafficking, primarily caused by the S1P1 receptor on lymphocytes, provides a new effective target for reducing inflammatory lesions in the CNS, as exemplified by FTY-720 (fingolimod), the first oral functional antagonist of S1P1 approved for alleviating relapsing–remitting MS [46]. FTY-720 has previously been shown to effectively reduce brain injury after cerebral haemorrhage in rodents and patients [17, 47–49]. Therefore, S1P1 regulation has attracted attention as a potential immunological intervention for TBI treatment [50]. However, whether FTY-720 can improve functional prognosis after TBI remains controversial [21]. Because the first-generation S1P receptor modulator FTY-720 is nonselective, activation of various S1P receptor isomers expressed in heart cells and vasculature may result in serious adverse effects, such as bradycardia and hypertension [51]. Studies have shown that the prophylactic application of FTY-720 has no effect on lesion size or neurological deficits in both acute and chronic head injury models and cannot stabilize the BBB or prevent neuronal apoptosis [21]. In addition, FTY-720 has been shown to induce and/or exacerbate vascular leakage in the lungs and brain by blocking the binding of S1P to S1P1[22, 52, 53]. Therefore, the longer half-life and nonselective receptor modulation of FTY-720 limit its application in TBI.

CYM-5442, a second-generation S1P1-selective agonist, has been reported to have a relatively short plasma half-life (3 h) after systemic injection, allowing rapid and preferential distribution to the brain [23], and thus exerts fewer effects on the patient’s blood pressure and heart rate. Previous studies have shown that CYM-5442 maintains EC barrier function through the S1P1 signalling pathway, restricts the escape of leukocytes from capillaries, and protects against inflammatory injury [54]. CYM-5442 has also been reported to mitigate the influenza virus-induced pulmonary cytokine storm and leukocyte infiltration [25]. Preclinical studies demonstrated a significant protective effect of CYM-5442 against inflammatory injury and the maintenance of EC barrier function, suggesting that CYM-5442 can successfully limit the pathological process of TBI. Due to the relatively short plasma half-life of CYM-5442, lymphocytopenia was induced at 3 h after administration, and the lymphocyte count returned to normal at 24 h after administration. Even daily administration of CYM-5442 (3 mg/kg) for 7 days did not result in persistent immunosuppression. Our experimental results are similar to those of previous results [24], revealing CYM-5442 can transiently suppress lymphocyte trafficking but does not result in persistent immunosuppression.

In addition to their effect on lymphocyte migration, S1P receptors are involved in the maintenance of the BBB. Although S1PR modulators can effectively reduce peripheral inflammation, some S1PR modulators do not significantly reduce EB extravasation or plasma escape from the BBB [21], suggesting that peripheral immunosuppression only partial explains the differential prognoses of patients with TBI. This may also explain why FTY-720 does not provide TBI protection. Previous studies have confirmed that CYM-5442 maintains EC barrier function through the S1P1 signalling pathway and protects against immune complex-induced vascular injury [54]. CYM-5442 has also been shown to maintain BBB integrity in ischaemic stroke by restricting vesicle transport [24]. In this study, we demonstrated that CYM-5442 significantly reduced EB extravasation and albumin escape from the BBB after TBI. Because EB/albumin crosses the BBB primarily by transcellular transport [9, 24], CYM-5442 may protect the BBB by inhibiting the vesicle transcytosis of cerebrovascular ECs after TBI. Mfsd2a is a key inhibitor of cerebrovascular EC vesicle transcytosis [12, 32, 40], and our experiments confirmed that Mfsd2a expression was decreased after TBI, which resulted in a decrease in the concentration of S1P in the ECM and a significant increase in vesicle transcytosis. Recent studies have shown that different concentrations of S1P in the brain ECM have different effects on BBB integrity. Mfsd2a deficiency causes insufficient transport of S1P in the brain parenchyma, resulting in increased vesicle transcytosis [22]. Our study showed that TBI downregulated Mfsd2a expression and that administration of the S1P1 agonist CYM-5442 significantly reversed the increase in vesicle transport caused by the decrease in the S1P concentration after Mfsd2a downregulation.

Of note, we cannot definitively conclude that the modulation of S1P1 on immune cells and vesicle transcytosis completely account for the mechanism by which CYM-5442 protects against TBI. This study focused on only the changes in the vesicle transport of cerebrovascular ECs induced by CYM-5442 after TBI, while previous experiments showed that an S1P1 agonist also protected the BBB through TJs [50, 55]. Since S1P1 is widely distributed on a variety of cells involved in the TBI process, other potential cellular targets beyond immune cells need to be further studied. Reactive astrocytes in the CNS may be a site for S1P1 modulators [50]. In addition, CYM-5442, via the attenuation of cytokine amplification mediated by S1P1 in ECs, may play a role in TBI protection [25, 56]. The current study does not provide sufficient information on the optimal time window for CYM-5442 regulation or the optimal dose to achieve the maximum beneficial effect after TBI, and further studies should be conducted in the future.

## Conclusions

In summary, we demonstrate that Mfsd2a expression is decreased after TBI, leading to a decrease in the concentration of S1P in the ECM. The selective S1P1 agonist CYM-5442 is sufficient to provide posttraumatic protection by reducing vascular endothelial vesicle transcytosis and inhibiting the inflammatory response. Therefore, the application of CYM-5442 may provide a new approach for the treatment of TBI.

## Supplementary Information

**Additional file 1: Figure S1**. Injection of Mfsd2a AAV change Mfsd2a expression. A Changes of Mfsd2a expression at different time points after injection with Mfsd2a overexpression virus (Mfsd2a adeno-associated virus [AAV]) using WB. n=4. B Mfsd2a protein expression at different time points after injection with Mfsd2a shRNA using WB. n = 4. C After EB injected through the tail, red EB fluorescence (Em: 680 nm) is enhanced in Mfsd2a shRNA 30d group mice. All data was analyzed by one-way ANOVA and Tukey’s multiple comparisons test, *P<0.05, **P<0.01 vs versus Sham mice, all values are presented as the mean ±SD.

**Additional file 2: Figure S2**. CYM-5442 decreased the degree of EB dye extravasation in the coronal slices of the brains in each group on days 1 and 3 after TBI. n=6/group.

## Data Availability

The data used and/or analyzed during the current study are available from the corresponding author on reasonable request.

## Abbreviations

TBI: Traumatic brain injury
S1P1: Sphingosine-1-phosphate receptor 1
BBB: Blood–brain barrier
Mfsd2a: Major facilitator superfamily domain-containing 2a
AAV: Adeno-associated virus
MRI: Magnetic resonance imaging
ELISA: Enzyme-linked immunosorbent assay
TEM: Transmission electron microscopy
WB: Western blot
S1P: Sphingosine-1-phosphate
NDS: Neurological deficit scores
TJ: Tight junction
ECs: Endothelial cells
GPCRs: G protein-coupled receptors
S1PR: Sphingosine 1-phosphate receptor
MS: Multiple sclerosis
CNS: Central nervous system
SPNS2: Spinster Homolog 2
DMSO: Dimethylsulfoxide
IF: Immunofluorescence
CCI: Controlled cortical impact
LSCI: Laser speckle contrast imaging
ROIs: Regions of interest
CBF: Cerebral blood flow
EB: Evans Blue
PVDF: Polyvinylidene difluoride
HRP: Horseradish peroxidase
DHA: Docosahexaenoic acid
ECM: Extracellular matrix
