# Supplementary figures and images for: Selective sphingosine-1-phosphate receptor 1 modulator attenuates blood–brain barrier disruption following traumatic brain injury by inhibiting vesicular transcytosis
Source: Fluids Barriers CNS. 2022 Jul 11;19:57. doi: 10.1186/s12987-022-00356-6 (PMC9277863; doi:10.1186/s12987-022-00356-6)

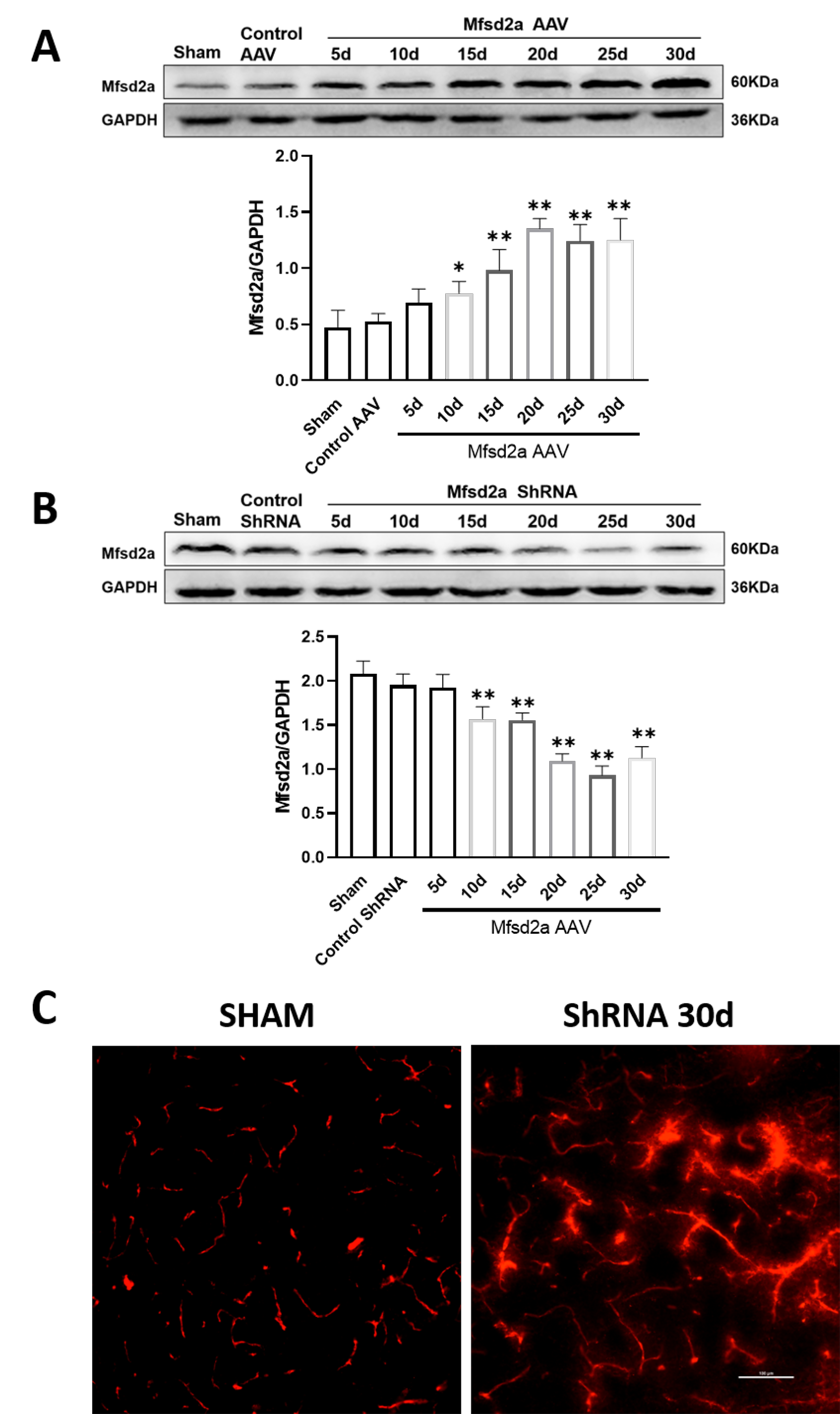

Supplement: Supplementary file 1 — Additional file 1: Figure S1. Injection of Mfsd2a AAV change Mfsd2a expression. A Changes of Mfsd2a expression at different time points after injection with Mfsd2a overexpression virus (Mfsd2a adeno-associated virus [AAV]) using WB. n=4. B Mfsd2a protein expression at different time points after injection with Mfsd2a shRNA using WB. n = 4. C After EB injected through the tail, red EB fluorescence (Em: 680 nm) is enhanced in Mfsd2a shRNA 30d group mice. All data was analyzed by one-way ANOVA and Tukey’s multiple comparisons test, *P<0.05, **P<0.01 vs versus Sham mice, all values are presented as the mean ±SD. [file 12987_2022_356_MOESM1_ESM.tif]

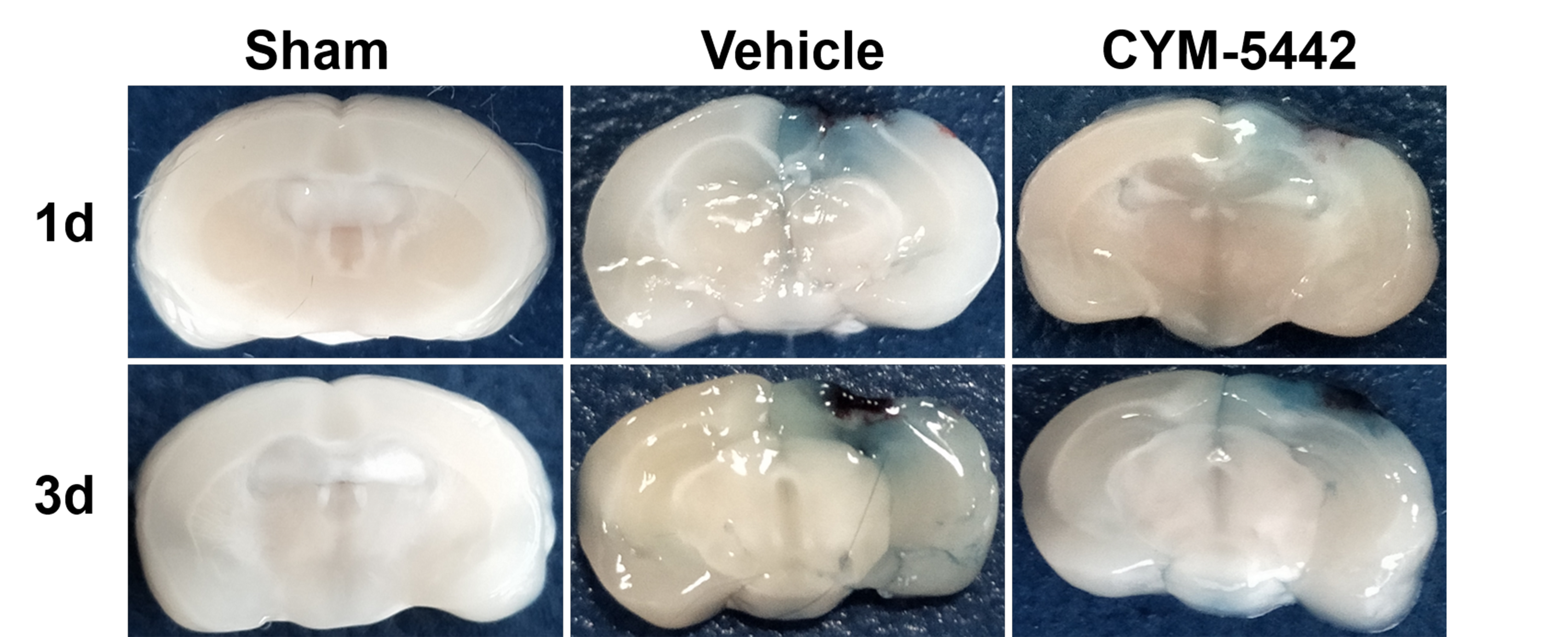

Supplement: Supplementary file 2 — Additional file 2: Figure S2. CYM-5442 decreased the degree of EB dye extravasation in the coronal slices of the brains in each group on days 1 and 3 after TBI. n=6/group. [file 12987_2022_356_MOESM2_ESM.tif]
